# Epithelioid hemangioendothelioma presenting as multiple pulmonary nodules following primary lung adenocarcinoma resection: a diagnostic challenge and clinical management dilemma

**DOI:** 10.1093/jscr/rjag212

**Published:** 2026-03-29

**Authors:** Ali Ahmad Korairi, Yeong Jeong Jeon

**Affiliations:** Thoracic Surgery Unit, Department of General Surgery, Prince Sultan Military Medical City, Abu Bakr Alrazi St, As Sulaimaniah Riyadh 12233, Saudi Arabia; Department of Thoracic and Cardiovascular Surgery, Samsung Medical Center, Sungkyunkwan University School of Medicine, Seoul, South Korea

**Keywords:** epithelioid hemangioendothelioma, lung adenocarcinoma, multiple primary malignancies, pulmonary nodules, differential diagnosis

## Abstract

New pulmonary nodules after curative lung cancer surgery are usually presumed metastatic, yet rare second primaries can mimic this pattern and radically change management. Pulmonary epithelioid hemangioendothelioma (EHE) is an ultra-rare vascular sarcoma that often presents as bilateral, small nodules radiologically indistinguishable from metastases. A 58-year-old never-smoker developed innumerable bilateral sub-centimeter nodules 9 months after thoracoscopic right middle lobectomy for pT1aN0 lung adenocarcinoma. Surgical biopsy of multiple nodules—performed alongside resection of a separate enlarging lesion—revealed EHE with lymphatic invasion. This case illustrates diagnostic challenges in post-surgical lung cancer surveillance and emphasizes the importance of tissue diagnosis when clinical presentation deviates from expected patterns. The coexistence of primary lung adenocarcinoma and pulmonary EHE is exceptionally rare. This case highlight on importance of maintaining a broad differential diagnosis for new pulmonary nodules in cancer survivors, particularly when radiological patterns are atypical. Histopathological confirmation remains essential for accurate diagnosis and optimal treatment planning.

## Introduction

The development of new pulmonary nodules following primary lung cancer treatment presents one of the most challenging diagnostic dilemmas in oncology practice. While metastatic disease represents the most probable diagnosis, clinicians must consider a spectrum of possibilities including inflammatory conditions, benign lesions, and second primary malignancies.

Epithelioid hemangioendothelioma (EHE) is an ultra-rare vascular neoplasm of intermediate malignant potential, with an estimated incidence of less than one in one million individuals. First described by Weiss and Enzinger in 1982, EHE exhibits distinctive histopathological features and unpredictable clinical behavior. Pulmonary EHE typically manifests as multiple bilateral nodules, creating a radiological appearance that can closely mimic metastatic disease. The coexistence of primary lung adenocarcinoma and pulmonary EHE in the same patient is extraordinarily rare, with only sporadic case were reported in the literature.

## Case presentation

A 58-year-old never-smoker with hypertension only presented after an incidental chest computed tomography (CT) identified a right middle lobe (RML) nodule. She was asymptomatic, and physical examination and routine laboratory tests were unremarkable.

**Figure 1:**
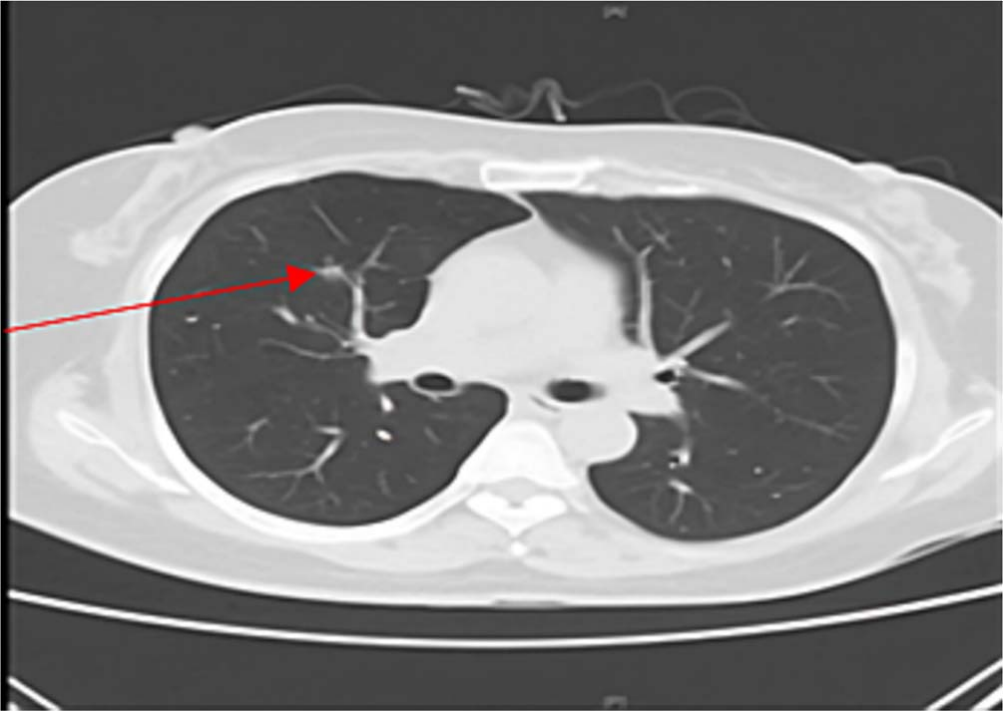
CT scan of the chest showing right middle lobe nodule.

Chest CT showed interval growth of the RML nodule to 14 mm with an increased solid component (solid portion 9.6 mm, Fig. 1). The multidisciplinary team recommended surgical intervention for that lesion due to its concerning features. The patient underwent thoracoscopic right middle lobectomy. Pathological examination of the RML specimen revealed well-differentiated adenocarcinoma with mixed lepidic and acinar patterns. The tumor measured 14 × 10 mm with a 6-mm invasive component. Importantly, there was no evidence of lymphovascular or perineural invasion, and all examined lymph nodes were negative. The final staging was pT1aN0M0.

**Figure 2:**
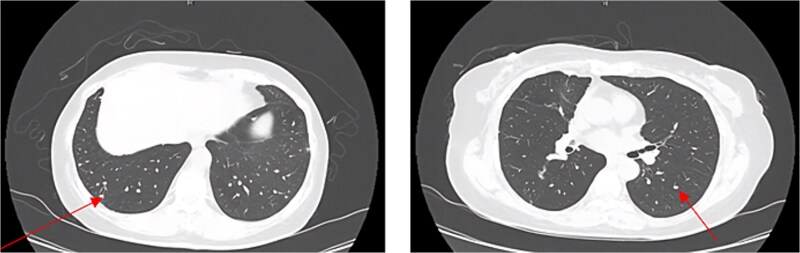
CT chest showing multiple bilateral nodule.

Surveillance chest CT scans were obtained at 3-month intervals after surgery. At 6 months post-surgery, multiple new small bilateral pulmonary nodules were identified. Follow-up chest CT at 9 months demonstrated persistence of the bilateral nodules with mild interval growth (Fig. 2). Positron emission tomography (PET)-CT revealed multiple subcentimeter pulmonary nodules with mild or no fluorodeoxyglucose uptake and was considered most compatible with pulmonary metastases.

The appearance of multiple bilateral nodules in the setting of recent lung adenocarcinoma resection created a significant diagnostic dilemma. The differential diagnosis included:

- **Intrapulmonary metastases** from the primary adenocarcinoma (most likely)
- **Granulomatous inflammation** (tuberculosis, histoplasmosis)
- **Sarcoidosis**
- **Second primary malignancy** (least likely)

The multidisciplinary team concluded that histopathologic confirmation was warranted. The patient underwent thoracoscopic diagnostic resection of multiple small nodules. Histopathological analysis yielded three separate masses (each 4 × 3 × 3 mm) revealed EHE with evidence of lymphatic invasion. The EHE diagnosis was confirmed through characteristic histopathological features including epithelioid endothelial cells with intracytoplasmic vacuoles within myxohyaline stroma. Immunohistochemical staining demonstrated positivity for endothelial markers (CD31, CD34, Factor VIII-related antigen).

## Discussion

Epithelioid hemangioendothelioma is a rare vascular neoplasm that presents significant diagnostic challenges, particularly when it occurs in the context of another primary malignancy [1]. The development of new pulmonary nodules in a patient with a history of malignancy typically raises immediate concern for metastatic disease. However, this case underscores the importance of maintaining a broad differential diagnosis and obtaining histopathological confirmation when the clinical presentation is atypical or unexpected.

EHE predominantly impacts the hepatic system, with the prevalence of pulmonary EHE being significantly lower than that of hepatic EHE. The majority of individuals diagnosed with pulmonary EHE remain asymptomatic or present with nonspecific respiratory manifestations, including cough, dyspnea, thoracic discomfort, and hemoptysis. Patients exhibiting pulmonary EHE are frequently identified incidentally through imaging techniques, as the findings from physical examinations tend to lack specificity [2].

Pulmonary EHE typically presents radiologically as multiple bilateral nodules, usually well-defined, non-calcified, and range in size from a few millimeters to several centimeters [3, 4]. In our patient, the radiological appearance of multiple small nodules was consistent with the typical presentation of pulmonary EHE. However, this finding was initially interpreted as a potential metastatic disease from the primary lung adenocarcinoma.

Histopathologically, EHE is characterized by epithelioid endothelial cells with intracytoplasmic vacuoles within a myxohyaline stroma [4, 5]. Immunohistochemically, these tumors typically express endothelial markers such as CD31, CD34, and factor VIII-related antigens [3, 5]. In our case, the histopathological examination of the resected nodules revealed the characteristic features of EHE, confirming the diagnosis of a second primary malignancy rather than metastatic disease.

The clinical behavior of pulmonary EHE is variable and unpredictable, with a 5-year survival rate ranging from 60% to 80% [6]. Factors associated with poor prognosis include the presence of pulmonary symptoms, pleural effusion, peripheral lymphadenopathy, and extensive intravascular, endobronchial, or interstitial tumor spread [7]. The optimal management strategy for pulmonary EHE remains undefined due to its rarity and the consequent lack of large clinical trials. Treatment options include surgical resection for localized disease, chemotherapy, radiation therapy, antiangiogenic agents, immunotherapy, and observation for asymptomatic patients with indolent disease [3, 5].

The coexistence of primary lung adenocarcinoma and pulmonary EHE in the same patient, as observed in our case, is exceptionally rare [8]. In our patient, the absence of a personal or family history of malignancy and the lack of exposure to known carcinogens suggest that the occurrence of these two distinct malignancies may be coincidental rather than causally related.

This case highlights several important clinical lessons. First, it emphasizes the critical importance of histopathological confirmation of suspected metastatic lesions, particularly with atypical clinical and radiological presentation. Second, it highlights the importance of a comprehensive approach to evaluating new pulmonary nodules in patients with a history of malignancy, including consideration of rare entities such as EHE. Finally, it underscores the significance of multidisciplinary collaboration in managing complex oncological cases.

## Conclusions

This case report demonstrates the critical importance of maintaining diagnostic vigilance and pursuing tissue confirmation when clinical presentations deviate from expected patterns in post-surgical cancer surveillance. The discovery of EHE as a second primary malignancy rather than metastatic disease fundamentally altered the patient’s treatment approach and prognosis.

## Data Availability

All data generated or analysed during this study are included in this published article. Additional inquiries can be directed to the corresponding author.

## References

[1] Huang H, Wang M, Zhu J. Malignant pulmonary epithelioid hemangioendothelioma masquerading as lung adenocarcinoma: a possible radiological and pathological diagnostic pitfall. Pulmonology 2024;30:681–4. 10.1016/j.pulmoe.2024.04.007 38702280

[2] Aung TT, Chu A, Kondapi D et al. A case of pulmonary epithelioid hemangioendothelioma with literature review. Case Rep Oncol Med 2020;2020:8048056. 33101745 10.1155/2020/8048056 PMC7568163

[3] Rosenberg A, Agulnik M. Epithelioid hemangioendothelioma: update on diagnosis and treatment. Curr Treat Options Oncol 2018;19:19. 10.1007/s11864-018-0536-y 29546487

[4] Young KE, Sung KT, Joungho H et al. Thoracic epithelioid hemangioendothelioma: imaging and pathologic features. Acta Radiol 2011;52:161–6. 10.1258/ar.2010.100292 21498344

[5] Tsuchihashi K, Baba E. Epithelioid hemangioendothelioma—its history, clinical features, molecular biology and current therapy. Jpn J Clin Oncol 2024;54:739–47. 10.1093/jjco/hyae037 38555494

[6] Chou CY, Hu HW, Chen TWW et al. A case of primary pleural epithelioid hemangioendothelioma achieving stable disease with paclitaxel treatment: a case report and literature review. Respirol Case Rep 2024;12:e01341. 10.1002/rcr2.1341 38559902 PMC10978069

[7] Semenisty V, Naroditsky I, Keidar Z et al. Pazopanib for metastatic pulmonary epithelioid hemangioendothelioma—a suitable treatment option: case report and review of anti-angiogenic treatment options. BMC Cancer 2015;15:402. 10.1186/s12885-015-1395-6 25967676 PMC4437555

[8] Jang YC, Hung WC, Su TC et al. Primary pulmonary epithelioid hemangioendothelioma. BMJ Case Rep 2023;16:e254915. 10.1136/bcr-2023-254915 PMC10503352 37709495

